# Socioeconomic inequality in any tobacco use versus current smoking across 66 DHS countries: Indirect implications for smokeless tobacco and oral cancer prevention

**DOI:** 10.18332/tid/226096

**Published:** 2026-08-31

**Authors:** Yin Tong, Hongtao Li, Xinyue Li, Mianjia Wan, Peng Zhou, Juncheng He, Kangwen Ming

**Affiliations:** 1 Department of Acupuncture and Rehabilitation, The Affiliated Traditional Chinese Medicine Hospital of Guangzhou Medical University, Guangzhou, China; 2 Guangzhou University of Chinese Medicine, Guangzhou, China; 3 Department of Stomatology, Guangdong Women and Children Hospital, Guangzhou, China

**Keywords:** smokeless tobacco, socioeconomic inequality, concentration index, demographic and health surveys, oral cancer

## Abstract

**INTRODUCTION:**

Smokeless tobacco use is a major preventable risk factor for lip and oral cavity cancer, with a particularly heavy burden in South and Southeast Asia. We aimed to quantify, across countries, whether the socioeconomic gradient of any tobacco use is more pro-poor than that of smoked tobacco alone, thereby indirectly assessing the additional inequality attributable to smokeless tobacco.

**METHODS:**

In this secondary analysis of publicly available Demographic and Health Surveys (DHS) data (66 countries, survey years 1999–2024), supplemented by the WHO Global Health Observatory and World Bank indicators, we computed concentration indices (CIXs), the slope index of inequality (SII), and Wagstaff-normalized CIXs for smoking and any tobacco use across wealth, education level, and residence dimensions. Eligible observations were country-survey estimates stratified by wealth quintile, education level, or urban/rural residence, with complete data across all subgroups; national-total, age-, and region-only estimates were excluded. The core hypothesis was tested indirectly, via paired comparison of any-tobacco versus smoked-only CIXs (81 paired observations), because DHS smokeless indicators are available only as national aggregates. Wagstaff decomposition quantified the relative contributions of socioeconomic determinants.

**RESULTS:**

The mean wealth-related CIX for smoking was -0.045 (SD=0.184), with 66% of estimates negative. The mean CIX for any tobacco use (-0.094) was significantly lower than for smoking alone (-0.060; paired ΔCIX= -0.033, 95% CI: -0.060 – -0.007, p=0.017), with 62% of pairs showing a more pro-poor gradient for any tobacco use. Education level and residence together accounted for >80% of explainable inequality in the decomposition analysis.

**CONCLUSIONS:**

The socioeconomic gradient of any tobacco use is significantly more pro-poor than that of smoking alone, suggesting that smokeless tobacco amplifies tobacco-related health inequality. These ecological findings suggest that tobacco-control monitoring may benefit from integrating smokeless tobacco into inequality surveillance; individual-level studies are needed before specific populations are targeted.

## INTRODUCTION

Lip and oral cavity cancer constitutes a substantial component of the global cancer burden. An estimated 370000 new cases occurred globally in 2019, with over 90% of the disease burden concentrated in South and Southeast Asia^1^. Approximately 30.8% of new oral cancer cases globally are attributable to smokeless tobacco and areca nut use, with 90.2% of attributable cases concentrated in low- and middle-income countries^2^. This evidence indicates that smokeless tobacco is a highly preventable but comparatively under-studied contributor to oral cancer, particularly with respect to its socioeconomic distribution.

Socioeconomic inequality in tobacco use is a classic topic in public health research. Smoking prevalence consistently exhibits a pro-poor gradient in low- and middle-income countries, with lower socioeconomic status groups showing significantly higher rates^3,4^. Sreeramareddy and Acharya^4^, analyzing DHS data from 22 Sub-Saharan African countries, found that absolute socioeconomic inequality in male smoking prevalence was approximately three times that observed among females. Dai et al.^5^ noted that smoking reduction has been substantially slower in low- and middle-income countries, leading to persistent inequalities in tobacco-attributable disease burden globally. Chen et al.^6^ found that low level of education and low income were significantly associated with dual and poly-tobacco use among males in 19 low- and middle-income countries.

However, the socioeconomic distribution of smokeless tobacco use may differ fundamentally from that of smoking. Smokeless tobacco products – particularly low-cost gutka and areca nut preparations – are highly accessible through street-level retail, are not subject to smoking bans, and are frequently regarded as cultural tradition^7^. Yang et al.^8^ reported a 4.4% smokeless tobacco use prevalence among adolescents aged 12–16 years across 138 countries. Spencer et al.^9^ demonstrated that tobacco tax increases produce the largest smoking reductions among the poorest 20% of the population. Vladisavljevic et al.^10^ and Macias Sanchez and Garcia Gomez^11^ confirmed the crowding-out effect of tobacco consumption on household expenditure for food, education, and healthcare, with some households falling below the poverty line. Collectively, this evidence suggests that the socioeconomic gradient of smokeless tobacco use may be stronger than that of smoking – a hypothesis not yet subjected to direct cross-national quantitative testing.

The existing literature presents several gaps. First, to our knowledge, no study has employed a unified inequality measurement framework – such as the concentration index or the slope index of inequality – to directly compare smoking and smokeless tobacco use across countries. Second, the relative contributions of structural factors driving tobacco inequality have not been quantitatively decomposed in a cross-national context. Third, it remains unknown whether inequality magnitude varies systematically by national income level or survey period. Addressing these gaps has direct policy relevance for formulating differentiated intervention strategies within the global tobacco control agenda^7,9^.

Because DHS does not release smokeless-tobacco estimates stratified by socioeconomic position, the smokeless gradient cannot be measured directly. We therefore infer it indirectly: because any-tobacco use comprises smoking plus smokeless use, a more pro-poor gradient for any-tobacco than for smoked-tobacco alone implies that the smokeless component is itself concentrated among the disadvantaged. Accordingly, this study aimed to: 1) estimate absolute and relative inequality indices for smoking and any tobacco use across multiple socioeconomic dimensions; 2) directly test whether the socioeconomic gradient of any tobacco use is more pro-poor than that of smoking alone; 3) apply Wagstaff decomposition to quantify the relative contributions of socioeconomic determinants; and 4) examine systematic variation in inequality by national income level and survey period.

## METHODS

### Study design and data sources

This was a secondary analysis of Demographic and Health Surveys (DHS) data, using the country-survey estimate as the unit of analysis; the study covered 66 countries with survey years spanning 1999–2024. DHS was selected as the primary source because it releases tobacco-use estimates pre-stratified by wealth quintile, education level, and residence in a harmonized format across many low- and middle-income countries – the structure required for cross-nationally comparable concentration-index estimation, as used in prior multi-country DHS inequality analyses^12^. Data were obtained from three publicly available sources: the DHS Program Indicator Data^13^ (current smoking, current smokeless tobacco use, and current any-tobacco use among adults, retrieved on 28 May 2026), the WHO Global Health Observatory^14^ (national adult smokeless-tobacco and smoking prevalence, from 2010 onward), and the World Bank Development Indicators^15^ (GDP per capita, Gini coefficient, urban population share, and health expenditure as % of GDP, 2010–2024). The exact source indicator codes and definitions are provided in Supplementary file Table S5. World Bank indicators were linked via ISO3 codes with a ±3-year matching tolerance. All data were aggregate-level, de-identified, publicly available secondary data.

### Study population and selection flow

DHS indicator records were retrieved for all available socioeconomic breakdowns and the DHS-preferred estimate per subgroup (full retrieval parameters in the Supplementary file), yielding 15677 initial records. We included country-survey estimates stratified by one of three socioeconomic dimensions – wealth quintile, educational attainment, or urban-rural residence – because these are the dimensions consistently released across DHS surveys and are the standard axes for equity monitoring. An estimate was eligible only if data were complete for every subgroup within a country-survey-dimension. National-total estimates and age- or region-based groupings were excluded, as they do not represent a socioeconomic gradient. The exact DHS field labels used for selection are listed in the Supplementary file.

**Figure 1:**
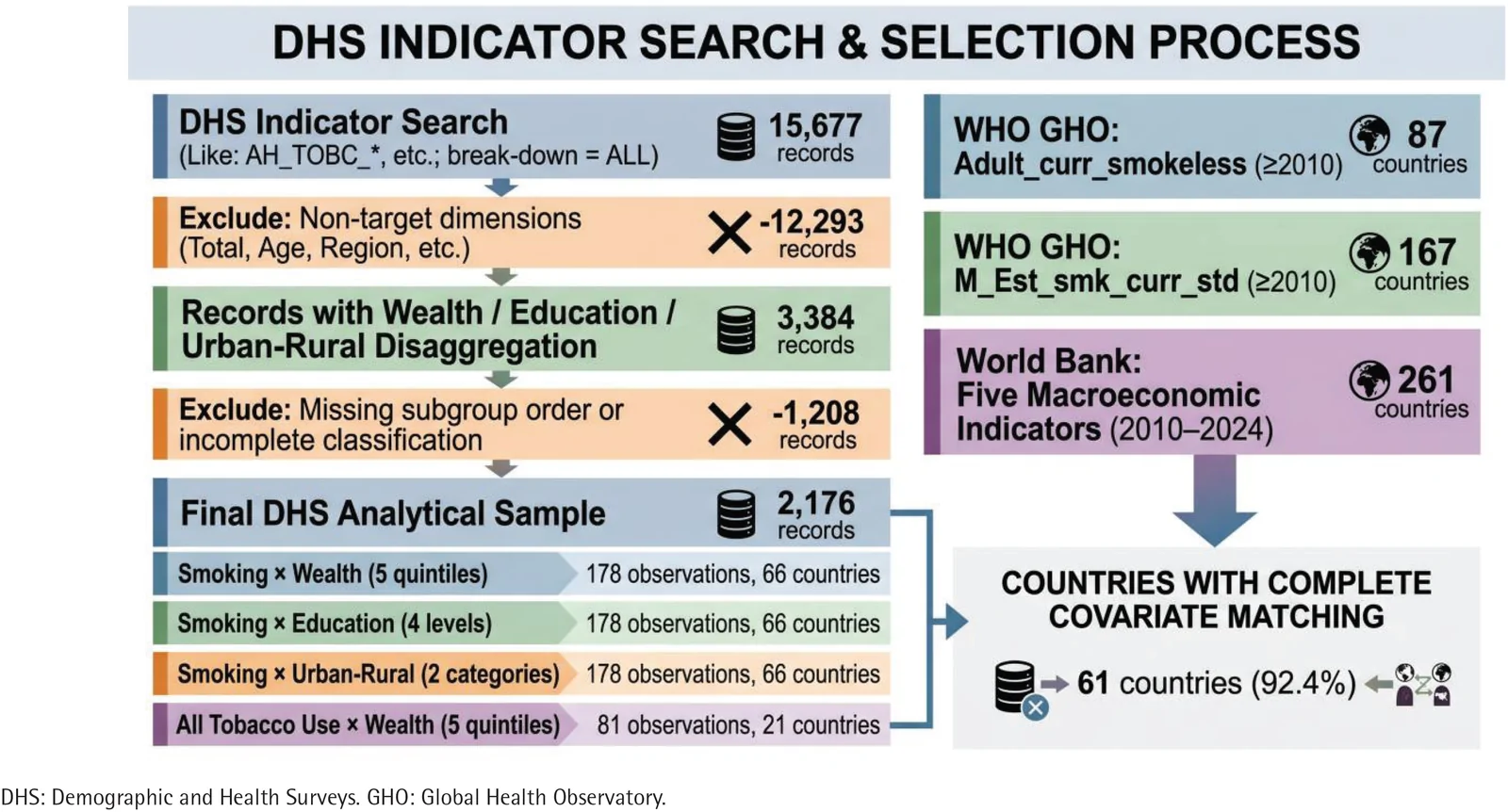
Study selection flowchart

Following selection (Figure 1), the final analytic sample comprised 178 country-survey observations with wealth-stratified smoking data (66 countries), 178 education-stratified observations (66 countries), and 178 residence-stratified observations (66 countries). Any-tobacco use wealth-stratified data (81 observations, 21 countries) were used for paired comparisons. Sex-stratified estimates were combined using weighted population denominators. Smokeless tobacco indicators (AH_TOBU_*_ASM), after applying the same criteria, contained only national aggregate data and were used solely for descriptive reporting.

### Variable definitions

Socioeconomic dimensions: wealth quintile (lowest, order=1, to highest, order=5); education level (no education, order=1; primary, 2; secondary, 3; higher, 4); and residence (rural, order=1; urban, order=2). lower order values indicate greater disadvantage. Outcome variables were subgroup weighted prevalence estimates (%) for the population aged 15–49 years. DHS estimates incorporate survey-specific sampling weights; denominator-weighted values served as subgroup population weights. Country-level covariates included log-GDP per capita, Gini coefficient, urban population share, and health expenditure as % of GDP.

### Missing data

The DHS indicator dataset contains only records with valid estimates; there were no missing estimates within subgroups. Among 66 DHS countries, 61 (92.4%) had complete data for all four World Bank covariates. Wagstaff decomposition was restricted to these 61 countries (complete-case analysis). Primary inequality indices did not depend on World Bank covariates and were unaffected by covariate missingness.

### Statistical analysis

*Inequality indices*

For each country-survey-dimension, subgroups were ranked by ascending socioeconomic position and ridit scores were computed using population shares as weights. The slope index of inequality (SII) was estimated via weighted least squares (WLS) regression (SII<0: concentration among disadvantaged groups). The relative index of inequality was computed as ŷ(R=1)/ŷ(R=0). The concentration index (CIX) was computed using the convenient regression approach, ranging from -1 to +1. The Wagstaff-normalized CIX^*^= CIX/(1−p) corrected for boundedness. Point estimates with 95% confidence intervals were reported.

*Decomposition analysis*

Wagstaff decomposition was performed on the wealth-related CIX for smoking in 61 countries with complete covariate data. Explanatory variables included education CIX, residence CIX, and four standardized country-level covariates, fitted via multiple linear regression. The relative contribution of each factor was |β_k × SD(X_k)| / Σ|β_j × SD(X_j)| × 100%.

*Stratified and sensitivity analyses*

Stratified analyses were performed by GDP per capita quartile and survey period (2010–2014 vs ≥2015). Paired t-tests compared any-tobacco versus smoked-only CIXs. Sensitivity analyses included: Wagstaff-normalized CIX substitution, restriction to the most recent survey per country, and extension of the covariate matching window to ±5 years.

*Software*

All analyses were performed in Python 3.12^16^ using *pandas*, *numpy*, *scipy*, and *scikit-learn*. The complete analysis code has been publicly archived (https://github.com/1947314628-ui/tobacco-inequality-dhs).

### Ethics

Each DHS survey received ethical approval from the relevant national institutional review board and obtained informed consent. This study exclusively used publicly available, aggregate-level, de-identified secondary data and is exempt from additional IRB review in accordance with the Declaration of Helsinki and CIOMS guidelines.

## RESULTS

### Sample characteristics

The analytic sample comprised DHS data from 66 countries (survey years 1999–2024). Observations per dimension: wealth, 178; education, 178; residence, 178. A total of 81 country-level paired observations were available for the core hypothesis test. Complete World Bank covariate data were available for 61 countries (Supplementary file Table S1).

### Socioeconomic inequality in smoking

*Wealth dimension*

The mean wealth-related CIX for smoking was -0.045 (SD=0.184); 118/178 (66%) of estimates were negative (p<0.001 for one-sample t-test against zero). The mean SII was -2.2 percentage points (SD=8.9). The mean Wagstaff-normalized CIX was -0.041.

**Figure 2:**
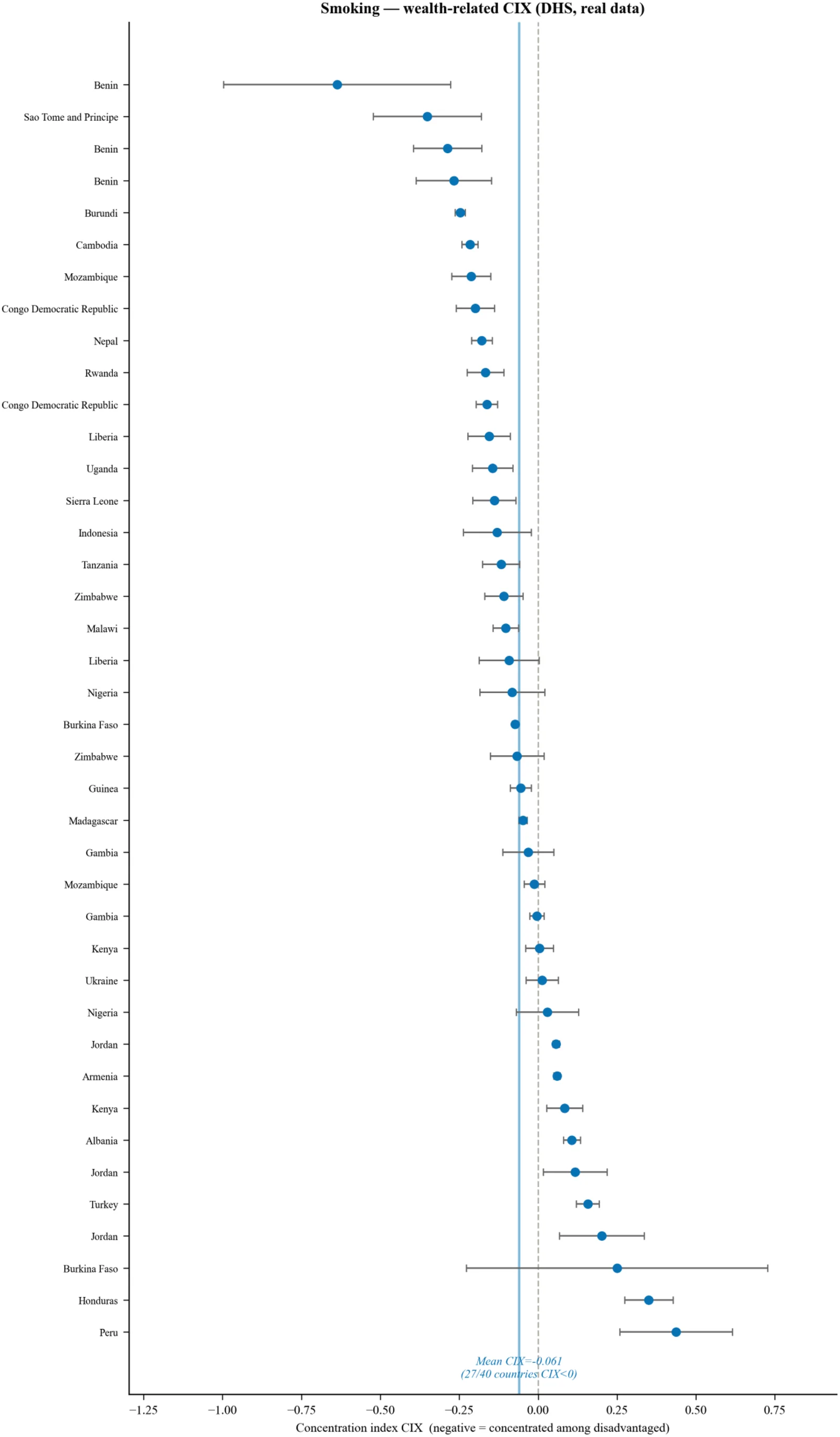
Country-level wealth-related concentration index (CIX) for current smoking, by country: forest plot of 178 country-survey observations from 66 Demographic and Health Survey (DHS) countries, 1999–2024. Points: CIX estimates; horizontal lines: 95% CIs. Dashed line: CIX=0; red line: pooled mean CIX. CIX<0 indicates concentration among poorer populations

Country-level wealth gradients showed poorer-group concentration for smoking and any tobacco use; these detailed gradient panels are shown in Supplementary file Figure S1. Country-level wealth-related CIX estimates and 95% CIs for current smoking are shown in Figure 2 (complete data: Supplementary file Table S2).

*Education and residence dimensions*

**Figure 3:**
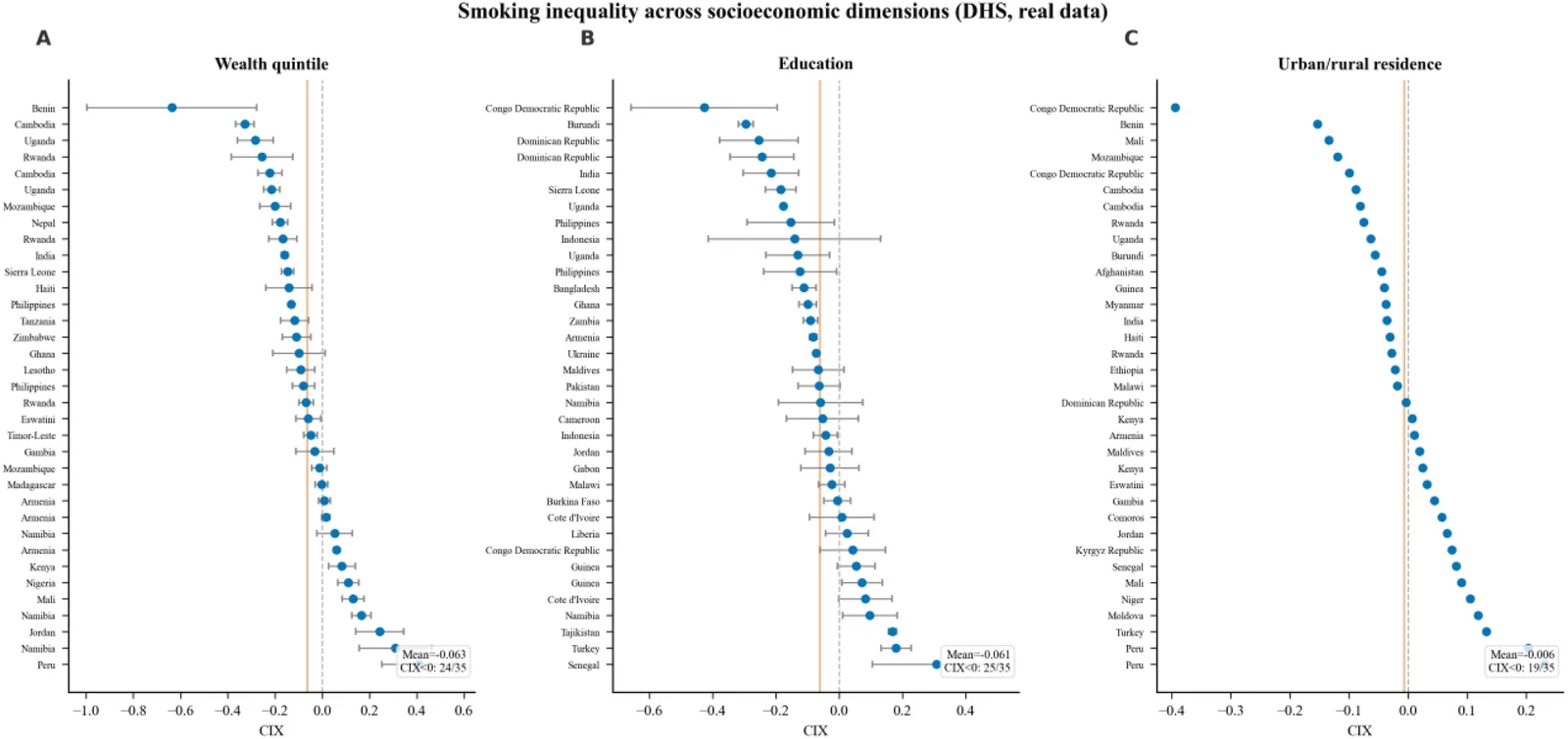
Smoking CIX across three socioeconomic dimensions in 178 country-survey observations from 66 DHS countries, 1999–2024: A) Wealth quintile; B) Education level; C) Urban/rural residence. Each point represents one country-survey estimate; red vertical lines show the mean CIX within each dimension

The mean education-related CIX for smoking was -0.050 (SD=0.141), with 70.8% of estimates negative. The mean education SII was -3.5 percentage points. The residence gradient was weaker: mean CIX=0.009 (SD=0.119; 51.7% negative). A comparison across the three dimensions is shown in Figures 3A–3C.

*Paired comparison: Any tobacco vs smoking*

In 81 paired observations, the mean CIX for any tobacco use was -0.094 (SD=0.113) versus -0.060 (SD=0.161) for smoking alone. The paired difference was -0.033 (95% CI: -0.060 – -0.007; p=0.017). In 62% of pairs, the CIX for any tobacco was smaller than for smoking alone, indicating a more pro-poor gradient when smokeless tobacco is included (Supplementary file Figure S2).

*Decomposition of wealth-related inequality*

In 61 countries with complete covariate data, six factors collectively explained 94.3% of the total variation in the wealth-related CIX for smoking (R^2^=0.943). Relative contributions: education, 41.1%; residence, 42.0%; GDP per capita, 1.2%; Gini coefficient, 3.1%; urbanization, 4.1%; and health expenditure, 8.4%. Education and residence together accounted for over 80% of explainable variation (Supplementary file Figures S3A and S3B).

*Stratified analysis by economic development*

**Figure 4:**
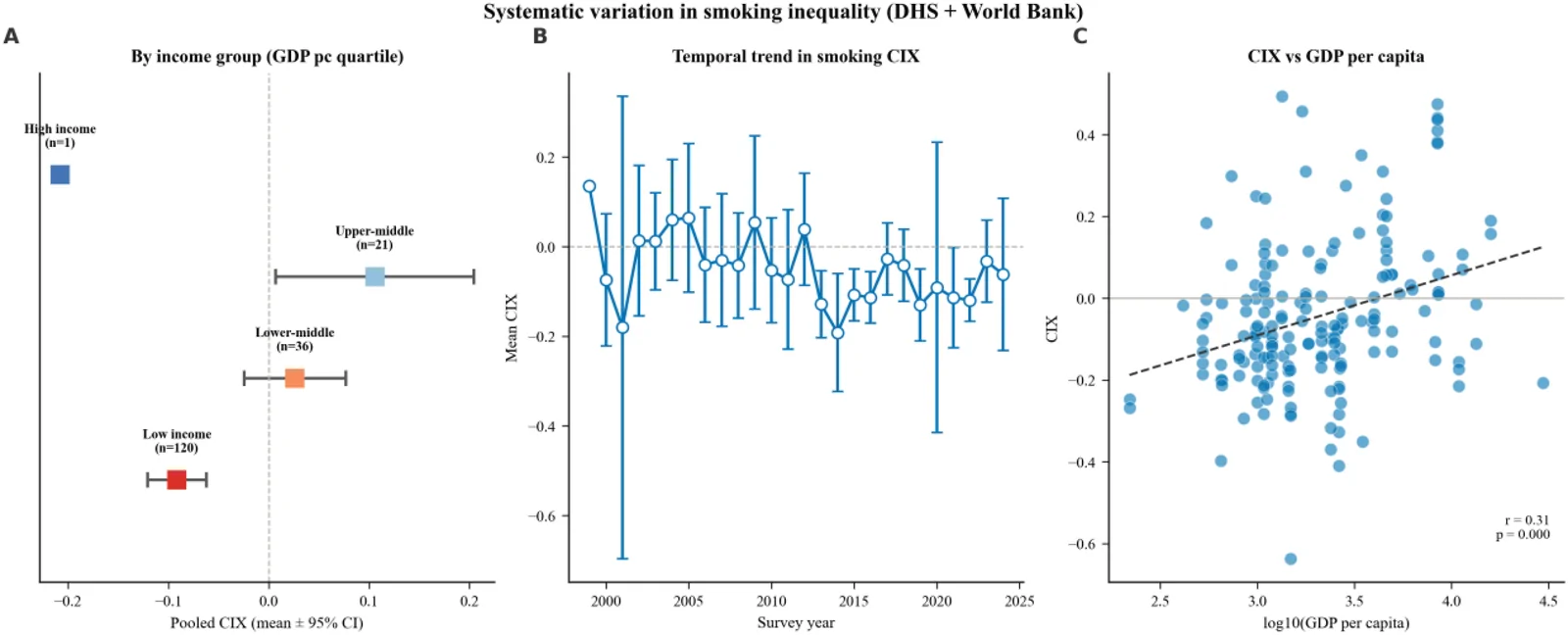
Systematic variation in smoking inequality across 66 DHS countries: A) Pooled mean CIX and 95% CI by GDP per capita quartile; B) Mean CIX by survey year period; C) CIX versus log-GDP per capita; dashed line indicates the fitted linear trend

Stratified by GDP per capita quartile: Low income (mean CIX= -0.091, n=120); Low middle (mean CIX=0.026, n=36); Upper middle (mean CIX=0.106, n=21); High income (mean CIX= -0.208, n=1). CIX magnitude decreased with increasing income level (Figure 4A). The mean CIX for surveys from 2015 onward (-0.082, n=59) was more negative than for 2010–2014 (-0.027, n=119; Figure 4B). The relationship between log-GDP and CIX is shown in Figure 4C.

### Global distribution of smokeless tobacco use

In WHO GHO data (87 countries), adult smokeless tobacco prevalence ranged from 0.0% to 38.3% (unweighted mean: 4.5%, SD=7.0). The five highest-burden countries were Palau (38.3%), Bangladesh (27.5%), Marshall Islands (21.6%), India (21.4%), and Afghanistan (19.3%; Supplementary file Figure S5). High-burden countries were concentrated in South/Southeast Asia and the Western Pacific (Supplementary file Table S3).

### Sensitivity analyses

The Wagstaff-normalized CIX was fully consistent with the standard CIX (Spearman ρ>0.99). Restricting to the most recent survey per country (n=66), the mean CIX was -0.058, closely approximating the primary estimate (-0.045). Covariate matching tolerance (±3 vs ±5 years) had a negligible impact on CIX estimates (<0.005; Supplementary file Figure S4 and Table S4).

## DISCUSSION

### Principal findings

Drawing on DHS data from 66 countries, this study offers one of the most geographically extensive cross-national analyses to date of socioeconomic inequality in tobacco use and, to our knowledge, the first cross-national paired test of whether smokeless tobacco imposes a stronger pro-poor gradient than smoking. Three principal findings emerged. First, smoking exhibited a robust pro-poor gradient. Second, the paired ΔCIX of -0.033 (p=0.017) supported the hypothesis that any tobacco use is more strongly concentrated among the disadvantaged than smoking alone. Third, education and residence were the dominant contributors to wealth-related inequality in decomposition analysis.

### Comparison with existing literature

The pro-poor gradient in smoking is consistent with multiple DHS- and Global Adult Tobacco Survey (GATS)-based studies^3,4,12^. Huang et al.^17^ identified socioeconomic inequalities in smoking and cessation behaviors among Chinese adults. Tanaka et al.^18^ reported widening inequality in Japan over 2001–2016. Disney et al.^19^ demonstrated that disadvantaged groups in Australia were being ‘left behind’ in tobacco control progress. Regarding smokeless tobacco, the Global Burden of Disease (GBD) 2019 Chewing Tobacco Collaborators^20^ noted no significant decline in Southeast Asia over 1990–2019. Ghate et al.^21^, Halder et al.^22^, Shaikh and Saikia^23^, and Chugh et al.^24^ all provided single-country evidence on smokeless tobacco inequalities, but none employed a unified cross-national framework. Bandyopadhyay and Irfan^25^ estimated education and wealth inequalities specifically in smokeless tobacco use across rural and urban Bangladesh and India, and Sreeramareddy and Harper^26^ documented persistent socioeconomic gradients in any-tobacco use in Nepal using DHS data; neither, however, provided a harmonized cross-national comparison of the smoked-versus-any-tobacco gradient. Carnazza et al.^27^ found substantial cross-country heterogeneity in smoking inequality across EU countries, consistent with the inter-country CIX variation observed here (SD=0.184).

### Interpretation of Wagstaff decomposition

Education and residence dominated the decomposition (>80% combined), while macroeconomic indicators played limited roles (1.2% for GDP, 3.1% for Gini). This aligns with Huang et al.^17^ and Halder et al.^22^ in indicating that individual-level socioeconomic position – rather than national macroeconomic indicators – drives inequality in health behaviors. Mann et al.^28^ similarly emphasized that tobacco control evaluation must incorporate equity dimensions.

### Policy implications

The core finding – a significantly stronger pro-poor gradient for any tobacco use versus smoking alone – suggests that current policies may systematically underestimate smokeless tobacco’s contribution to health inequality. Puljevic et al.^29^ noted that smokeless tobacco occupies a marginalized position in endgame strategies. Mills et al.^30^ called for systematic equity incorporation into tobacco control. Spencer et al.^9^ demonstrated that tobacco tax increases yield pro-poor health benefits, yet Vladisavljevic et al.^10^ and Macias Sanchez and Garcia Gomez^11^ showed that crowding-out effects are more severe among low-income households. Price and taxation policies targeting smokeless tobacco must therefore be implemented alongside cessation support and alternative livelihood programs.

### Strengths and limitations

Strengths of the study include use of publicly accessible databases with publicly archived analysis code (full reproducibility); simultaneous reporting of absolute and relative inequality indices per WHO monitoring guidelines; a paired comparison design controlling for country-specific confounders; and geographical coverage (66 DHS countries) exceeding prior single-country or single-region studies.

Limitations of the study include: 1) DHS smokeless tobacco indicators lack wealth, education, or residence stratification – the paired comparison of any-tobacco versus smoked-only CIXs provides only indirect inference; 2) the ecological design precludes individual-level causal inference; 3) heterogeneity across DHS surveys in questionnaire design and definitions^3^; 4) aggregate-level covariates in the decomposition cannot fully substitute for individual-level pathway analyses^31,32^; and 5) the ±3-year World Bank covariate matching may introduce measurement error, though sensitivity analyses confirmed no material effect on conclusions.

### Future research

Future research should: 1) advocate for strengthened stratified data collection and release for smokeless tobacco indicators in DHS and other national surveys; 2) combine DHS microdata within multilevel frameworks^33^; 3) directly decompose the any-tobacco CIX into smoking and smokeless tobacco components once stratified data become available; and 4) investigate whether smokeless tobacco-attributable oral cancer burden is disproportionately distributed across socioeconomic groups^34^.

## CONCLUSIONS

This study, based on empirical data from 66 DHS countries, provides systematic cross-national evidence on socioeconomic inequality in tobacco use and, to our knowledge, presents the first cross-national assessment of an additional pro-poor amplification associated with smokeless tobacco. Education and residence are the core structural drivers. The global tobacco control agenda should incorporate smokeless tobacco into inequality monitoring frameworks and prioritize disadvantaged rural populations with low level of education – a strategy with profound significance for reducing the global socioeconomic inequality burden of lip and oral cavity cancer.

## Supplementary Material



## Data Availability

The data supporting this research are available from the following publicly accessible sources: The DHS Program (https://dhsprogram.com), WHO GHO (https://www.who.int/data/gho), and the World Bank (https://data.worldbank.org).
