# Supplementary material for: Socioeconomic inequality in any tobacco use versus current smoking across 66 DHS countries: Indirect implications for smokeless tobacco and oral cancer prevention
Source: Tob Induc Dis. 2026 Aug 31;24:10.18332/tid/226096. doi: 10.18332/tid/226096 (PMC13532050; doi:10.18332/tid/226096)
Supplement: Supplementary file 1 [file TID-24-152-s1.pdf]

Tobacco Induced Diseases

Supplementary file

© 2026 Tong Y. et al.

DOI: 10.18332/tid/226096

The content has been provided by the author(s) and has not been reviewed, verified, or endorsed by European Publishing. It may not have undergone peer review. The views, opinions, and recommendations expressed are solely those of the author(s) and do not necessarily reflect the position of European Publishing. European Publishing accepts no responsibility or liability for any consequences arising from the use of, or reliance on, this content.

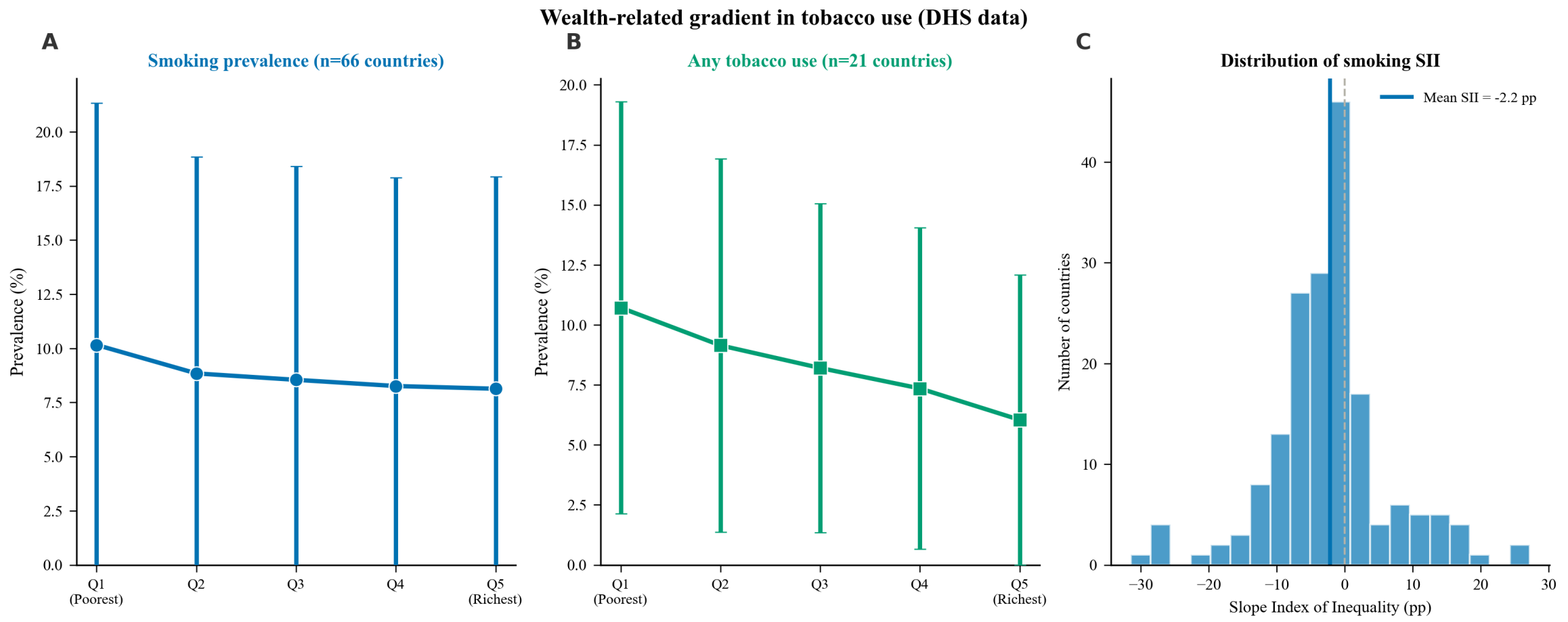

Figure S1. Wealth-related gradient in tobacco use in DHS data. (A) Country-specific smoking prevalence trajectories across wealth quintiles and mean gradient. (B) Mean gradient for any tobacco use. (C) Distribution of country-level smoking SII estimates.

Figure S2. Paired comparison of wealth-related CI for any tobacco vs. smoking alone

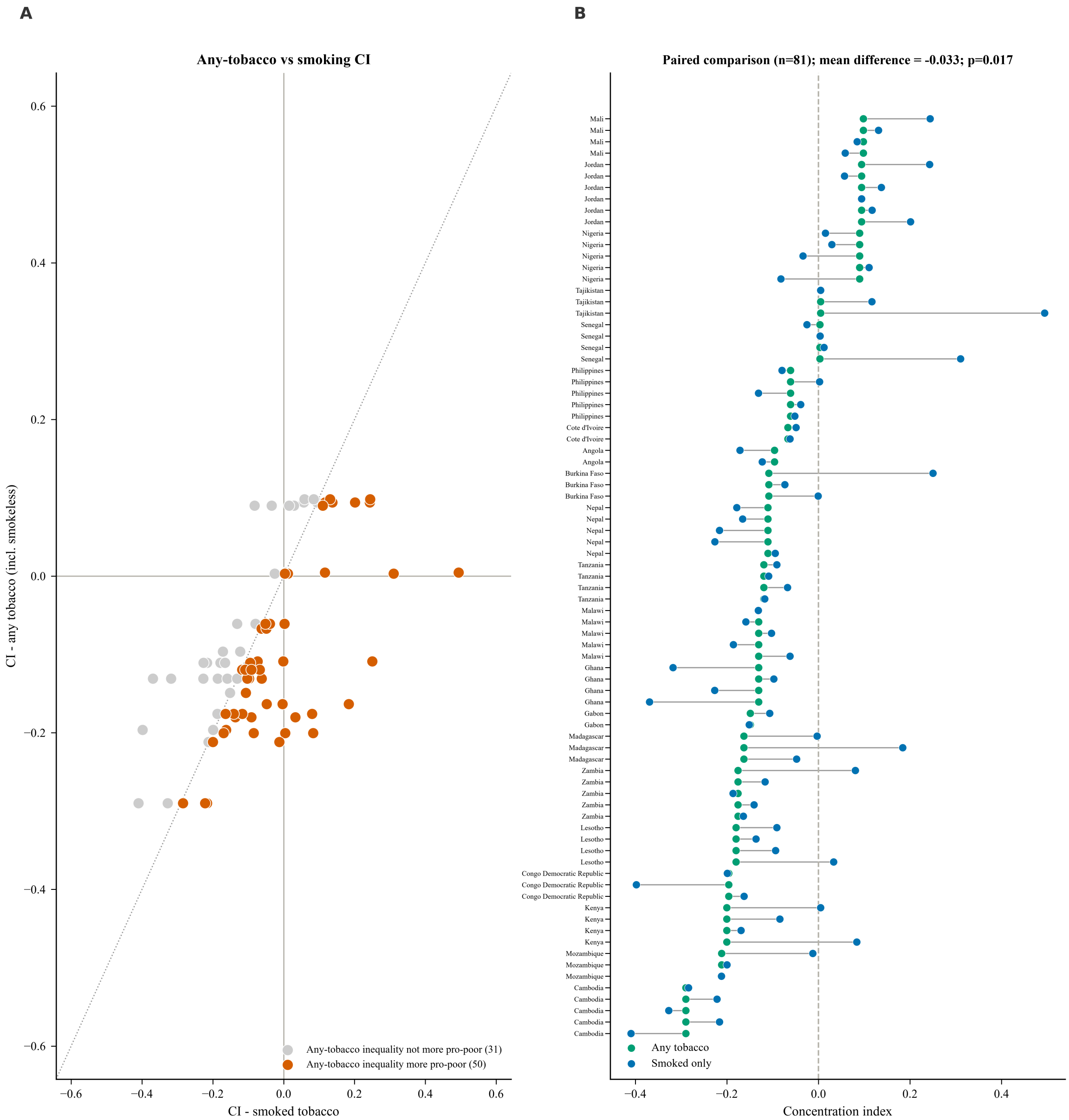

Figure S2. Paired comparison of wealth-related CIX for any tobacco versus smoking alone. Panel B was regenerated with expanded vertical spacing and smaller y-axis labels to improve readability.

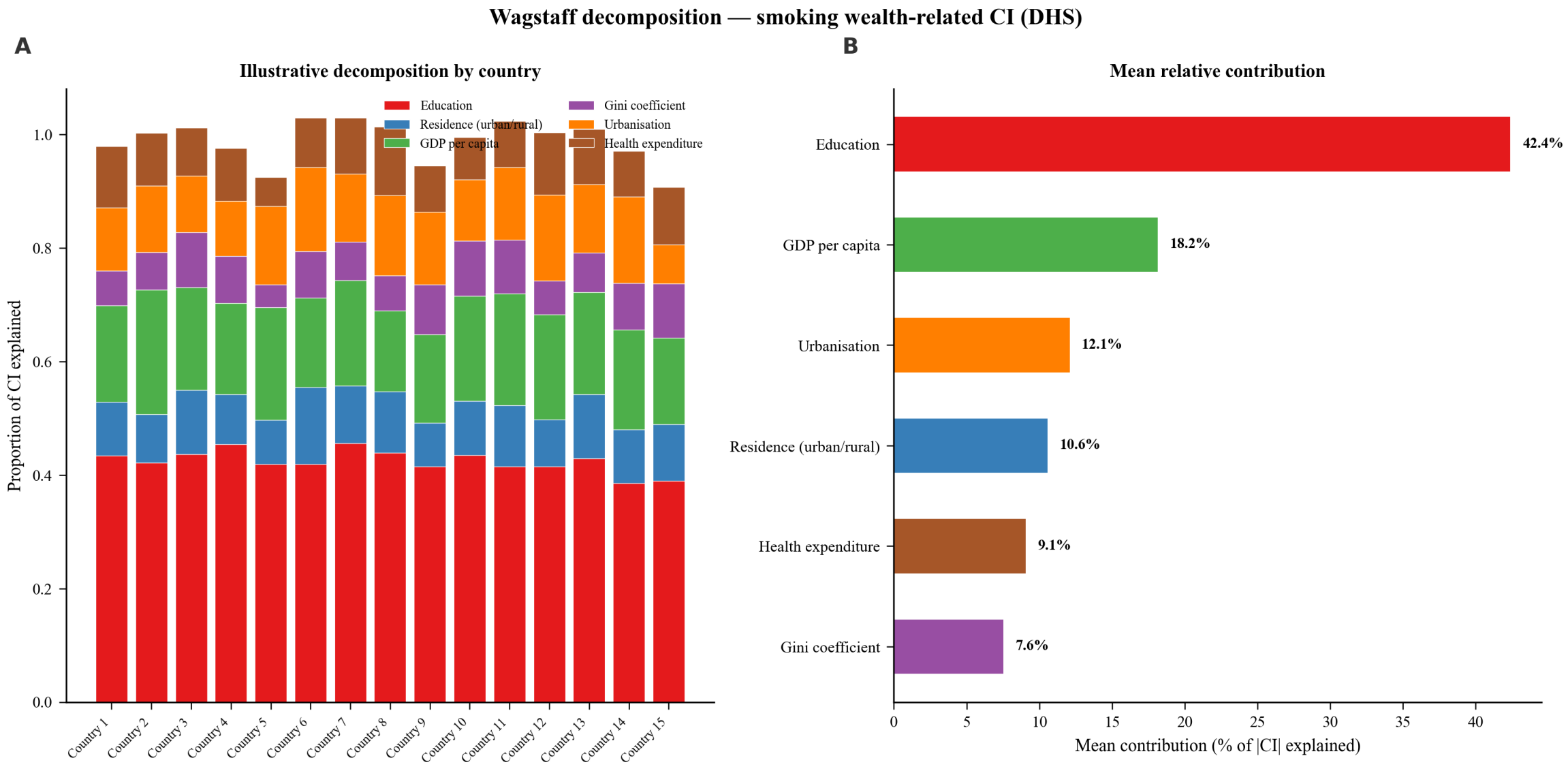

Figure S3. Wagstaff decomposition of wealth-related CIX for smoking. (A) Country-level schematic. (B) Mean relative contribution.

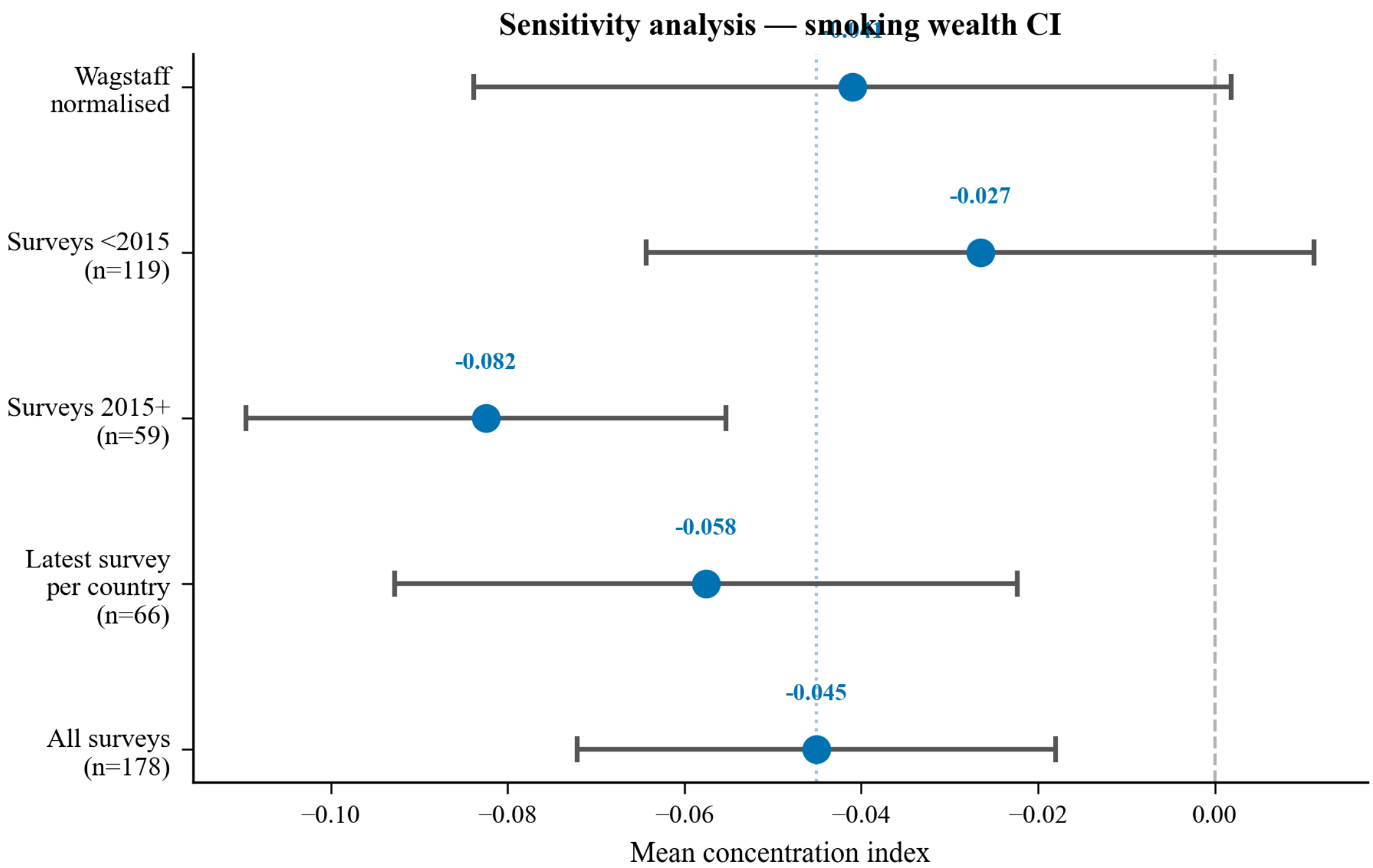

Figure S4. Sensitivity analysis: mean CIX for smoking under alternative analytical scenarios.

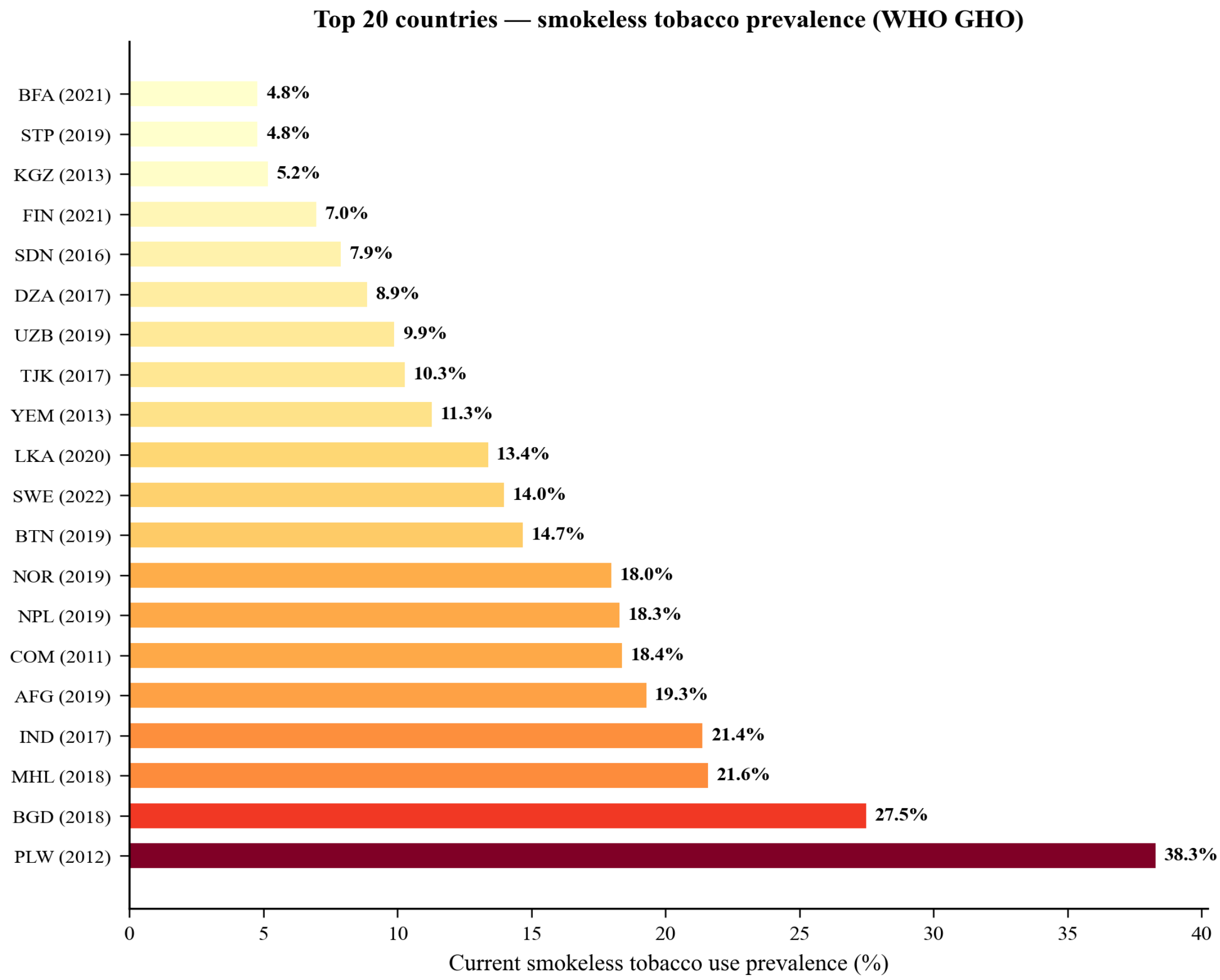

Figure S5. Top 20 countries by adult smokeless tobacco prevalence in WHO Global Health Observatory data, both sexes, most recent available year. Labels show full country names and data year; color intensity corresponds to prevalence level.

Table S1. Descriptive statistics of DHS countries included in the analysis

Country	N surveys	Mean prevalence (%)	Subgroup range (%)	Latest year	GDP per capita (USD)
Bangladesh	2	57.8	35.0-75.1	2007	2593
Azerbaijan	1	48.5	41.5-57.3	2006	7284
Papua New Guinea	1	36.9	29.4-40.7	2017	3007
Armenia	4	27.6	10.9-68.9	2016	8556
Ukraine	1	26.6	22.4-31.2	2007	5389
Turkey	2	25.0	11.4-45.4	2008	15893
Timor-Leste	2	18.0	13.2-22.7	2016	1332
Moldova	1	16.4	12.9-23.0	2005	7576
Jordan	6	15.9	6.3-28.8	2023	4618
Nepal	5	15.8	6.5-39.5	2022	1447
Guyana	1	15.1	7.0-25.9	2009	29675
Albania	2	14.9	6.6-28.4	2017	11378
Maldives	2	14.2	8.5-21.4	2016	13379
India	3	14.1	3.3-37.8	2020	2695
Myanmar	1	13.6	6.5-20.9	2016	1359
Lesotho	4	13.0	4.6-49.4	2023	972
South Africa	1	12.4	9.2-16.2	2016	6267
Afghanistan	1	11.7	6.9-18.0	2015	414
Kyrgyz Republic	1	11.0	0.0-16.4	2012	2420
Sierra Leone	3	10.7	4.8-19.2	2019	807
Namibia	3	10.2	3.2-19.6	2013	4413
Gabon	2	10.0	5.5-15.9	2019	8230
Madagascar	3	9.5	3.7-14.5	2021	545
Bolivia	1	8.7	5.3-16.0	2008	4421
Dominican Republic	3	8.6	3.3-21.6	2013	10876

**Eswatini**	1	8.6	6.6-14.2	2006	3910
**Pakistan**	2	8.6	4.6-13.4	2017	1479
**Philippines**	5	8.4	3.4-30.1	2022	3985
**Congo**	1	8.2	5.4-11.7	2011	2482
**Zambia**	5	8.1	2.4-14.8	2024	1187
**Niger**	2	7.8	0.0-21.3	2012	735
**Burundi**	2	7.2	0.9-20.0	2016	219
**Guatemala**	1	7.1	4.7-8.2	2015	6150
**Zimbabwe**	4	7.1	0.3-13.7	2015	2497
**Cambodia**	5	7.1	0.0-19.6	2021	2628
**Cote d'Ivoire**	2	7.1	4.6-10.7	2021	2728
**Comoros**	1	6.7	3.6-11.3	2012	1663
**Kenya**	4	6.3	2.7-10.1	2022	2132
**Gambia**	2	5.5	3.7-7.9	2019	871
**Mauritania**	1	5.4	3.9-6.6	2020	2110
**Nicaragua**	1	5.1	2.0-9.4	2001	2848
**Haiti**	4	5.1	1.5-10.7	2016	2143
**Congo Democratic Republic**	3	4.9	0.1-10.7	2023	649
**Mozambique**	3	4.9	2.2-17.6	2022	657
**Angola**	2	4.9	3.1-8.1	2023	2666
**Liberia**	3	4.8	1.2-10.6	2019	851
**Honduras**	2	4.7	0.6-8.7	2011	3426
**Peru**	6	4.7	0.7-14.5	2012	8452
**Rwanda**	5	4.5	0.9-11.1	2019	1000
**Uganda**	4	4.2	1.1-8.5	2016	1078
**Sao Tome and Principe**	1	3.9	1.2-9.3	2008	3491
**Malawi**	5	3.8	1.1-6.0	2024	523
**Tanzania**	4	3.8	1.7-6.2	2022	1187
**Burkina Faso**	3	3.7	0.0-8.4	2021	982
**Mali**	4	3.4	0.0-7.2	2023	1095
**Togo**	1	3.3	1.5-5.0	2013	1119
**Ethiopia**	3	3.1	1.3-6.8	2016	1134
**Senegal**	4	2.8	0.0-5.4	2023	1773
**Cameroon**	3	2.7	0.5-5.7	2018	1830
**Ghana**	4	2.7	0.4-7.5	2022	2391
**Indonesia**	4	2.5	1.0-8.1	2017	4925
**Guinea**	2	2.4	0.0-5.0	2018	1695
**Nigeria**	5	2.3	0.9-5.5	2024	1084
**Benin**	4	1.4	0.0-5.3	2017	1485
**Tajikistan**	3	0.9	0.0-2.9	2023	1341
**Egypt**	1	0.6	0.3-1.1	2005	3338

Table S2. Wealth-related inequality indices for tobacco smoking (all country-survey observations)

Country	Year	CI	SE	SII (pp)	RII
**Benin**	2001	-0.6371	0.1836	-0.57	-0.33
**Cambodia**	2005	-0.4099	0.1000	-9.36	-0.12
**Congo Democratic Republic**	2007	-0.3983	0.0626	-1.92	-0.11
**Ghana**	2014	-0.3693	0.0610	-3.34	-0.07
**Sao Tome and Principe**	2008	-0.3509	0.0874	-7.24	-0.05
**Cambodia**	2000	-0.3275	0.0195	-8.41	-0.01
**Ghana**	2008	-0.3180	0.1266	-6.05	0.00
**Liberia**	2013	-0.2946	0.0328	-6.11	0.04
**Benin**	2017	-0.2871	0.0553	-5.38	0.05
**Cambodia**	2021	-0.2842	0.0539	-13.90	0.06
**Uganda**	2011	-0.2834	0.0390	-6.40	0.06

**Benin**	2006	-0.2829	0.0843	-0.34	0.06
**Burundi**	2016	-0.2685	0.0596	-6.92	0.09
**Benin**	2012	-0.2673	0.0610	-3.84	0.09
**India**	2020	-0.2564	0.0205	-13.02	0.11
**Rwanda**	2019	-0.2550	0.0662	-4.17	0.11
**Togo**	2013	-0.2475	0.0505	-5.03	0.13
**Burundi**	2010	-0.2470	0.0081	-18.68	0.13
**Ghana**	2003	-0.2271	0.0609	-5.25	0.17
**Nepal**	2011	-0.2264	0.0500	-20.90	0.17
**Cambodia**	2014	-0.2220	0.0258	-12.63	0.18
**Uganda**	2006	-0.2195	0.0347	-7.93	0.19
**Cambodia**	2010	-0.2164	0.0131	-16.44	0.19
**Nepal**	2006	-0.2164	0.0369	-26.74	0.19
**Dominican Republic**	2013	-0.2145	0.0632	-8.54	0.20
**Uganda**	2016	-0.2143	0.0176	-3.56	0.20
**Mozambique**	2022	-0.2123	0.0316	-5.40	0.20
**Guyana**	2009	-0.2077	0.0567	-18.53	0.21
**Ethiopia**	2005	-0.2075	0.0674	-4.74	0.21
**Rwanda**	2015	-0.2018	0.0425	-4.67	0.23
**Mozambique**	2011	-0.1999	0.0335	-7.59	0.23
**Congo Democratic Republic**	2023	-0.1994	0.0311	-8.06	0.23
**Sierra Leone**	2013	-0.1893	0.0277	-12.86	0.26
**Zambia**	2013	-0.1871	0.0402	-10.50	0.26
**Malawi**	2010	-0.1862	0.0156	-4.93	0.26
**Nepal**	2001	-0.1787	0.0166	-31.50	0.28
**Pakistan**	2012	-0.1755	0.0210	-10.04	0.29
**Dominican Republic**	2007	-0.1738	0.0426	-9.24	0.30
**Angola**	2015	-0.1718	0.0478	-6.00	0.30
**Kenya**	2022	-0.1695	0.0318	-5.70	0.31
**Rwanda**	2000	-0.1671	0.0299	-5.17	0.31
**India**	2015	-0.1665	0.0147	-7.66	0.32
**Nepal**	2016	-0.1658	0.0584	-11.35	0.32
**Zambia**	2024	-0.1642	0.0122	-8.60	0.32
**Congo Democratic Republic**	2013	-0.1626	0.0173	-8.14	0.33
**India**	2006	-0.1591	0.0064	-26.41	0.34
**Malawi**	2015	-0.1589	0.0251	-3.26	0.34
**Dominican Republic**	2002	-0.1563	0.0148	-8.46	0.34
**Liberia**	2007	-0.1556	0.0344	-7.41	0.35
**Gabon**	2019	-0.1516	0.0131	-8.52	0.36
**Sierra Leone**	2019	-0.1464	0.0129	-7.14	0.37
**Ethiopia**	2016	-0.1454	0.0605	-2.09	0.37
**Haiti**	2006	-0.1450	0.0800	-3.35	0.37
**Uganda**	2000	-0.1447	0.0326	-4.97	0.38
**Myanmar**	2016	-0.1417	0.0282	-12.02	0.39
**Haiti**	2000	-0.1409	0.0499	-5.27	0.39
**Zambia**	2018	-0.1408	0.0199	-7.81	0.39
**Sierra Leone**	2008	-0.1390	0.0348	-12.85	0.39
**Congo**	2011	-0.1383	0.0179	-7.03	0.40
**Lesotho**	2023	-0.1365	0.0231	-13.71	0.40
**Malawi**	2024	-0.1315	0.0285	-3.05	0.42
**Philippines**	2003	-0.1314	0.0043	-15.01	0.42
**Indonesia**	2012	-0.1298	0.0550	-1.90	0.42
**Pakistan**	2017	-0.1248	0.0458	-6.53	0.44
**Angola**	2023	-0.1229	0.0511	-3.00	0.44
**Rwanda**	2010	-0.1179	0.0357	-3.50	0.46
**Tanzania**	2004	-0.1176	0.0301	-3.52	0.46
**Zambia**	2007	-0.1170	0.0377	-8.09	0.46
**Maldives**	2016	-0.1114	0.0155	-11.82	0.48
**Cameroon**	2018	-0.1110	0.0224	-1.95	0.48
**Cameroon**	2011	-0.1098	0.0333	-3.07	0.49
**Zimbabwe**	2015	-0.1089	0.0307	-5.50	0.49
**Tanzania**	2010	-0.1089	0.0211	-3.05	0.49

**Gabon**	2012	-0.1068	0.0340	-6.95	0.50
**Haiti**	2012	-0.1048	0.0345	-3.74	0.51
**Malawi**	2004	-0.1030	0.0209	-2.73	0.51
**Ghana**	2022	-0.0979	0.0566	-1.19	0.53
**Zimbabwe**	2010	-0.0970	0.0275	-5.76	0.53
**Nepal**	2022	-0.0945	0.0305	-6.19	0.54
**Lesotho**	2009	-0.0938	0.0414	-5.84	0.54
**Liberia**	2019	-0.0918	0.0485	-1.94	0.55
**Lesotho**	2014	-0.0913	0.0303	-6.96	0.55
**Tanzania**	2022	-0.0913	0.0268	-1.80	0.55
**Kenya**	2014	-0.0847	0.0195	-4.17	0.58
**Nigeria**	2003	-0.0822	0.0524	-1.08	0.59
**Indonesia**	2017	-0.0808	0.0327	-1.10	0.60
**Philippines**	2008	-0.0798	0.0242	-2.64	0.60
**Bangladesh**	2007	-0.0757	0.0095	-28.47	0.62
**Bangladesh**	2004	-0.0740	0.0110	-26.73	0.62
**Burkina Faso**	2021	-0.0737	0.0040	-2.12	0.62
**Haiti**	2016	-0.0687	0.0421	-1.79	0.65
**Rwanda**	2005	-0.0681	0.0157	-2.88	0.65
**Tanzania**	2015	-0.0676	0.0239	-1.36	0.65
**Zimbabwe**	2005	-0.0668	0.0436	-4.16	0.65
**Cote d'Ivoire**	2012	-0.0624	0.0361	-3.19	0.67
**Malawi**	2000	-0.0623	0.0401	-1.60	0.67
**Eswatini**	2006	-0.0591	0.0270	-2.94	0.69
**Guinea**	2018	-0.0558	0.0171	-1.34	0.70
**Honduras**	2011	-0.0553	0.0349	-2.32	0.71
**Philippines**	2022	-0.0517	0.0184	-1.39	0.72
**Cote d'Ivoire**	2021	-0.0490	0.0328	-1.73	0.73
**Timor-Leste**	2016	-0.0490	0.0146	-4.90	0.73
**Madagascar**	2021	-0.0478	0.0067	-3.05	0.74
**Timor-Leste**	2009	-0.0437	0.0075	-5.22	0.76
**Philippines**	2013	-0.0391	0.0200	-1.40	0.78
**Nigeria**	2008	-0.0341	0.0251	-0.72	0.81
**Gambia**	2013	-0.0318	0.0411	-1.10	0.82
**Azerbaijan**	2006	-0.0311	0.0233	-9.54	0.82
**Senegal**	2010	-0.0254	0.0545	-0.64	0.85
**Afghanistan**	2015	-0.0177	0.0443	-1.27	0.90
**Maldives**	2009	-0.0149	0.0324	-1.10	0.91
**Mozambique**	2003	-0.0126	0.0167	-0.29	0.92
**Ethiopia**	2011	-0.0115	0.0490	-0.23	0.93
**Papua New Guinea**	2017	-0.0104	0.0102	-2.42	0.94
**Comoros**	2012	-0.0101	0.0443	-0.42	0.94
**Gambia**	2019	-0.0043	0.0116	-0.15	0.97
**Madagascar**	2008	-0.0034	0.0137	-0.21	0.98
**Burkina Faso**	2010	-0.0010	0.0194	-0.04	0.99
**Philippines**	2017	0.0021	0.0296	0.07	1.01
**Senegal**	2023	0.0032	0.0509	0.06	1.02
**Kenya**	2008	0.0044	0.0224	0.15	1.03
**Tajikistan**	2023	0.0045	0.0559	0.06	1.03
**Armenia**	2016	0.0088	0.0128	1.10	1.06
**Senegal**	2017	0.0118	0.0464	0.29	1.08
**Ukraine**	2007	0.0118	0.0261	1.98	1.08
**Nigeria**	2018	0.0151	0.0543	0.14	1.10
**Armenia**	2005	0.0151	0.0197	1.16	1.10
**Armenia**	2000	0.0153	0.0087	6.22	1.10
**South Africa**	2016	0.0216	0.0173	1.71	1.14
**Nigeria**	2013	0.0290	0.0502	0.47	1.20
**Guatemala**	2015	0.0319	0.0174	1.44	1.22
**Lesotho**	2004	0.0328	0.0127	1.09	1.23
**Namibia**	2013	0.0522	0.0380	3.00	1.39
**Jordan**	2017	0.0568	0.0053	7.68	1.43
**Indonesia**	2007	0.0578	0.0144	0.95	1.44
**Indonesia**	2003	0.0579	0.0367	0.69	1.44
**Mali**	2012	0.0584	0.0289	1.63	1.45
**Armenia**	2010	0.0597	0.0052	5.47	1.46
**Albania**	2017	0.0700	0.0109	6.08	1.56
**Mauritania**	2020	0.0742	0.0373	2.55	1.61
**Zambia**	2002	0.0803	0.0887	1.79	1.67

**Niger**	2012	0.0811	0.0440	7.75	1.69
**Kenya**	2003	0.0834	0.0291	3.71	1.71
**Mali**	2018	0.0845	0.0382	2.07	1.72
**Jordan**	2023	0.0941	0.0171	13.90	1.83
**Moldova**	2005	0.1042	0.0424	10.90	1.97
**Albania**	2008	0.1067	0.0138	10.04	2.00
**Nigeria**	2024	0.1103	0.0225	1.29	2.05
**Cameroon**	2004	0.1151	0.0471	0.58	2.13
**Kyrgyz Republic**	2012	0.1162	0.0311	8.88	2.14
**Tajikistan**	2017	0.1166	0.0416	0.35	2.15
**Jordan**	2002	0.1169	0.0515	7.22	2.15
**Mali**	2023	0.1311	0.0236	3.38	2.39
**Zimbabwe**	1999	0.1353	0.1038	0.51	2.47
**Jordan**	2012	0.1375	0.0536	15.44	2.51
**Turkey**	2003	0.1570	0.0184	27.20	2.94
**Egypt**	2005	0.1599	0.1284	0.58	3.00
**Namibia**	2006	0.1658	0.0209	11.09	3.17
**Madagascar**	2004	0.1839	0.0272	8.51	3.72
**Turkey**	2008	0.1897	0.0192	26.64	3.93
**Jordan**	2007	0.2012	0.0686	16.46	4.39
**Bolivia**	2008	0.2038	0.0672	11.03	4.54
**Jordan**	2009	0.2429	0.0517	18.19	7.31
**Mali**	2006	0.2441	0.2765	0.21	7.46
**Burkina Faso**	2003	0.2499	0.2436	0.14	8.24
**Nicaragua**	2001	0.2752	0.0431	9.03	13.61
**Niger**	2006	0.2988	0.3014	0.19	29.42
**Namibia**	2000	0.3098	0.0784	19.50	70.74
**Senegal**	2005	0.3103	0.1342	0.80	71.02
**Honduras**	2005	0.3502	0.0392	4.97	-21.34
**Peru**	2007	0.3791	0.0682	16.63	-10.23
**Peru**	2004	0.3822	0.0577	12.28	-10.89
**Peru**	2010	0.4103	0.0804	14.96	-8.03
**Peru**	2011	0.4369	0.0912	14.10	-6.44
**Peru**	2009	0.4420	0.1000	16.50	-6.20
**Guinea**	2005	0.4566	0.2323	2.23	-5.68
**Peru**	2012	0.4749	0.1240	14.62	-5.10
**Tajikistan**	2012	0.4940	0.1575	0.50	-4.68

Table S3. Top 15 countries by adult current smokeless tobacco use prevalence (WHO GHO)

Country	Prevalence (%)	Year
**PLW**	38.3	2012
**BGD**	27.5	2018
**MHL**	21.6	2018
**IND**	21.4	2017
**AFG**	19.3	2019
**COM**	18.4	2011
**NPL**	18.3	2019
**NOR**	18.0	2019
**BTN**	14.7	2019
**SWE**	14.0	2022
**LKA**	13.4	2020
**YEM**	11.3	2013
**TJK**	10.3	2017
**UZB**	9.9	2019
**DZA**	8.9	2017

Table S4. Sensitivity analysis of wealth-related CI for tobacco smoking

Scenario	N	Mean CI	Mean SII (pp)
All surveys	178	-0.0451	-2.20
Latest survey per country	66	-0.0576	-2.22
Surveys 2015 and later	59	-0.0824	-3.09
Surveys before 2015	119	-0.0265	-1.76

Table S5. Paired comparison of wealth-related CI: all-tobacco use vs. smoked tobacco only

Country	CI_all_tobacco	CI_smoked	Delta CI
Tajikistan	0.0045	0.4940	-0.4895
Burkina Faso	-0.1088	0.2499	-0.3588
Madagascar	-0.1633	0.1839	-0.3472
Senegal	0.0031	0.3103	-0.3072
Kenya	-0.2004	0.0834	-0.2838
Zambia	-0.1760	0.0803	-0.2563
Lesotho	-0.1803	0.0328	-0.2131
Kenya	-0.2004	0.0044	-0.2048
Mozambique	-0.2117	-0.0126	-0.1991
Madagascar	-0.1633	-0.0034	-0.1599
Jordan	0.0941	0.2429	-0.1489
Mali	0.0981	0.2441	-0.1460
Kenya	-0.2004	-0.0847	-0.1157
Madagascar	-0.1633	-0.0478	-0.1155
Tajikistan	0.0045	0.1166	-0.1120
Burkina Faso	-0.1088	-0.0010	-0.1078
Jordan	0.0941	0.2012	-0.1072
Lesotho	-0.1803	-0.0913	-0.0890
Lesotho	-0.1803	-0.0938	-0.0865
Cambodia	-0.2900	-0.2164	-0.0736
Malawi	-0.1308	-0.0623	-0.0685
Cambodia	-0.2900	-0.2220	-0.0681
Philippines	-0.0610	0.0021	-0.0631
Zambia	-0.1760	-0.1170	-0.0590
Tanzania	-0.1195	-0.0676	-0.0518
Lesotho	-0.1803	-0.1365	-0.0438
Jordan	0.0941	0.1375	-0.0434
Gabon	-0.1490	-0.1068	-0.0422
Zambia	-0.1760	-0.1408	-0.0351
Burkina Faso	-0.1088	-0.0737	-0.0351
Congo Democratic Republic	-0.1963	-0.1626	-0.0338
Mali	0.0981	0.1311	-0.0330
Ghana	-0.1309	-0.0979	-0.0330
Kenya	-0.2004	-0.1695	-0.0309
Tanzania	-0.1195	-0.0913	-0.0282
Malawi	-0.1308	-0.1030	-0.0278
Jordan	0.0941	0.1169	-0.0228
Philippines	-0.0610	-0.0391	-0.0219

Nigeria	0.0899	0.1103	-0.0204
Cote d'Ivoire	-0.0671	-0.0490	-0.0181
Nepal	-0.1106	-0.0945	-0.0160
Zambia	-0.1760	-0.1642	-0.0118
Mozambique	-0.2117	-0.1999	-0.0118
Tanzania	-0.1195	-0.1089	-0.0106
Philippines	-0.0610	-0.0517	-0.0093
Senegal	0.0031	0.0118	-0.0087
Cambodia	-0.2900	-0.2842	-0.0058
Cote d'Ivoire	-0.0671	-0.0624	-0.0047
Tanzania	-0.1195	-0.1176	-0.0019
Senegal	0.0031	0.0032	-0.0001
Jordan	0.0941	0.0941	0.0000
Tajikistan	0.0045	0.0045	0.0000
Mozambique	-0.2117	-0.2123	0.0006
Malawi	-0.1308	-0.1315	0.0007
Gabon	-0.1490	-0.1516	0.0026
Congo Democratic Republic	-0.1963	-0.1994	0.0031
Zambia	-0.1760	-0.1871	0.0111
Mali	0.0981	0.0845	0.0136
Philippines	-0.0610	-0.0798	0.0188
Angola	-0.0964	-0.1229	0.0265
Malawi	-0.1308	-0.1589	0.0281
Senegal	0.0031	-0.0254	0.0285
Jordan	0.0941	0.0568	0.0373
Cambodia	-0.2900	-0.3275	0.0375
Mali	0.0981	0.0584	0.0397
Nepal	-0.1106	-0.1658	0.0552
Malawi	-0.1308	-0.1862	0.0554
Nigeria	0.0899	0.0290	0.0609
Nepal	-0.1106	-0.1787	0.0682
Philippines	-0.0610	-0.1314	0.0704
Nigeria	0.0899	0.0151	0.0748
Angola	-0.0964	-0.1718	0.0754
Ghana	-0.1309	-0.2271	0.0962
Nepal	-0.1106	-0.2164	0.1058
Nepal	-0.1106	-0.2264	0.1158
Cambodia	-0.2900	-0.4099	0.1199
Nigeria	0.0899	-0.0341	0.1240
Nigeria	0.0899	-0.0822	0.1721
Ghana	-0.1309	-0.3180	0.1872
Congo Democratic Republic	-0.1963	-0.3983	0.2019
Ghana	-0.1309	-0.3693	0.2384
